# Fault Diagnosis Method for Converter Stations Based on Fault Area Identification and Evidence Information Fusion

**DOI:** 10.3390/s24227321

**Published:** 2024-11-16

**Authors:** Shuzheng Wang, Xiaoqi Wang, Xuchao Ren, Ye Wang, Sudi Xu, Yaming Ge, Jiahao He

**Affiliations:** 1 Electric Power Engineering, Nanjing Institute of Technology, Nanjing 211167, Chinay00450230516@njit.edu.cn (J.H.); 2 State Grid Jiangsu Electric Power Co., Ltd., Nanjing 210024, China; 3 State Grid Jiangsu Electric Power Co., Ltd., Research Insititute, Nanjing 211103, China

**Keywords:** sequential event recording, real-time digital simulation, fuzzy Petri net, BP neural network, evidence information acquisition, Dempster–Shafer evidence theory

## Abstract

DC converter stations have a high voltage level, a long transmission distance, and complex internal equipment, and contain power electronic devices, which seriously endanger the stable operation of the system itself and the active distribution network at the receiving end when faults occur. Accurate fault analysis and diagnosis are critical to the safe and stable operation of power systems. Traditional fault diagnosis methods often rely on a single source of information, leading to issues such as insufficient information utilization and incomplete diagnostic scope when applied to DC transmission systems. To address these problems, a fault diagnosis method for converter stations based on preliminary identification of the fault range and the fusion of evidence information of the switch signal and electrical quantity is proposed. First, the preprocessing of converter station sequential event recording (SER) events and a statistical analysis of event characteristics are completed to initially determine the range of the fault.Then, a fuzzy Petri net model and a BP neural network model are constructed on the basis of the fault data from a real-time digital simulation system (RTDS), and the corresponding evidence information of the switch signal and electrical quantity are obtained via iterative inference and deep learning methods. Finally, on the basis of D-S evidence theory, a comprehensive diagnosis result is obtained by fusing the switch and electric evidence information. Taking the fault data of a DC converter station as an example, the proposed method is analyzed and compared with the traditional method, which is based on single information. The results show that the proposed method can reliably and accurately identify fault points in the protected area of the converter station.

## 1. Introduction

A high-voltage direct current (HVDC) transmission system has the characteristics of flexible control and low line loss and is an important technology for solving the problem of long-distance large-capacity power transmission [1,2,3]. In recent years, high-voltage DC transmission technology has been widely used in long-distance large-capacity power transmission projects [4,5,6]. The DC transmission system usually undertakes important tasks such as long-distance large-capacity transmission or grid interconnection. The main grid obtains electrical energy from the DC transmission system and distributes it to the active distribution network, which contains distributed power sources and flexible loads. The system structure is complex and the state is difficult to control. Therefore, comprehensive and efficient fault diagnosis methods for DC converter stations are urgently needed [7,8].

At present, the existing methods for DC fault diagnosis mainly include methods such as neural networks, expert systems, support vector machines, etc. The studies above, on the other hand, mostly looked at the single information source on electrical quantity in the fault recording. They did not consider the DC transmission system sequential event recording (SER), which has a lot of fault information, or the switch signal information, which shows how the control and protection devices are working [9,10,11]. Wang et al. used a parallel convolutional neural network (PCNN) to achieve the fault diagnosis of multiterminal DC transmission lines on the basis of fault amplitude features and fault frequency features of signal waveforms [12]. Chen et al. obtained the singular spectrum entropy of each layer through wavelet multiscale decomposition to obtain feature vectors for support vector machine training and testing, and on the basis of many simulations, it was verified that this method can accurately distinguish line faults [13]. Li et al. extracted fault features from the measured recorded waveform data of a substation with four types of faults and completed the construction and training of an integrated learning system model, which proved that the method has a low error rate and strong robustness [14]. Hu et al. carried out feature extraction for six kinds of faults, such as single-phase grounding, two-phase grounding, and DC line grounding on the AC side, and applied the improved support vector machine (SVM) to classify the faults for training, established a system fault identification model, and verified the accuracy and validity of the model for the identification of the six kinds of faults [15]. Li et al. used wavelet packet transform and principal component analysis (PCA) to detect and identify ground faults with low transition resistance occurring in high-voltage DC transmission systems [16]. Liu et al. achieved real-time and effective diagnosis of the causes of commutation failure in high-voltage DC transmission systems via a wavelet neural network approach [17]. Lin et al. performed real-time monitoring of parameter changes in high-voltage capacitors in DC filters via a parameter identification method to achieve the fault identification of DC filters in DC transmission systems [18].

Different faults within the same DC protection subregion may have similar electrical waveform characteristics. If the above research methods are applied to DC transmission systems, only some faults with large differences in electrical waveform can be distinguished in a small range. The problem of being unable to reliably and accurately identify faults in the protection subregion exists. It is necessary to make comprehensive use of multi-source information to improve the reliability and comprehensiveness of diagnosis. This paper proposes a fault identification and localization method for DC converter stations that is based on multi-source information fusion, with a focus on key data such as SER events, electric waveforms, and switch signals. The feasibility and accuracy of the proposed method are verified through an analysis of actual operation data of the project via an analysis of calculation examples.

The main contributions of this paper can be summarized as follows:

1. A preprocessing method of converter station SER data and a preliminary identification method of the converter station fault range are proposed to obtain the scope of the pending fault point.
2. A method for obtaining the evidence information of the switch signal and electrical quantity in converter stations is proposed, and the evidence information is obtained by constructing fuzzy Petri nets and training BP neural networks, respectively.
3. Fusion of the switch evidence information and electric evidence information of the converter station is achieved, and accurate identification of the fault point of the converter station is completed according to the fusion results. Moreover, the accuracy and applicability of the multievidence information fusion diagnosis method are verified through a comparison between the information fusion diagnosis results and the single-evidence information diagnosis results.
4. Some traditional diagnostic methods are compared with the method proposed in this paper. The results show that the proposed method can obtain more accurate and reliable fault diagnosis results than other methods can achieve.

The rest of this paper is organized as follows. The “Methods” section introduces the fault diagnosis methods of the converter station and explains the relevant theories involved. In the “Experiments and Results” section, a series of experiments are constructed, and the results and analysis details are given. Finally, our conclusions are drawn in the “Discussion” and “Conclusion” sections.

## 2. Methods

**Figure 1:**
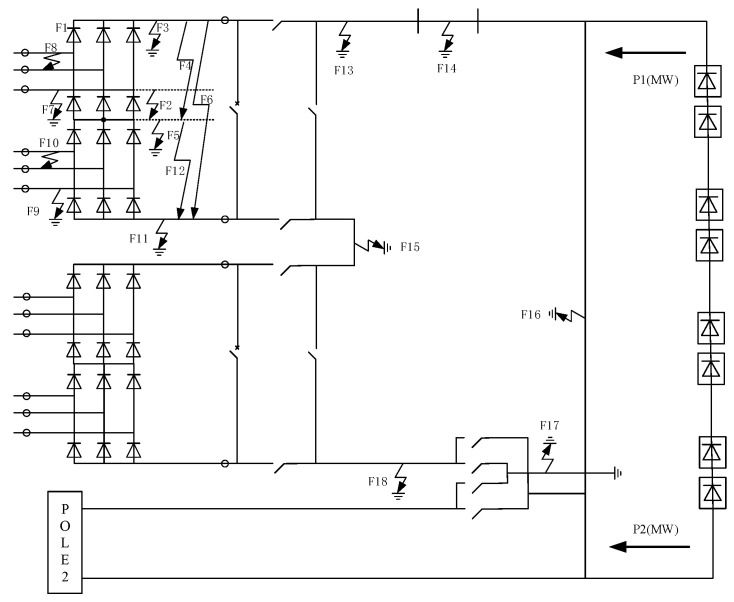
Converter station fault point diagram.

A direct current transmission system is usually divided into a converter area, a polar area, a bipolar area, a filter area, etc. A schematic diagram of the fault points is shown in Figure 1. The main faults in the converter area include pulse loss of the converter valve (F1), short-circuit of the converter and its bridge arm (F2, F4, F6, F12), DC-side grounding faults (F3, F5, F11), AC-side phase-to-phase short-circuit (F8, F10), and grounding faults (F7, F9). Polar area faults mainly include pole bus grounding (F13), pole neutral bus grounding (F18), pole midpoint grounding (F15), and DC line grounding (F14). The main faults in the bipolar area are bipolar neutral bus grounding (F17) and metallic return grounding (F16). The electrical waveforms corresponding to these fault points may have similar characteristics, while traditional DC system diagnostic methods yield better results in diagnosing faults with obvious differences in characteristics in a small area, such as grounding faults and line faults. If the diagnostic scope covers the entire HVDC transmission system, the traditional method considers only a single source of information, and the results may have problems such as missed judgments and incorrect judgments.

**Figure 2:**
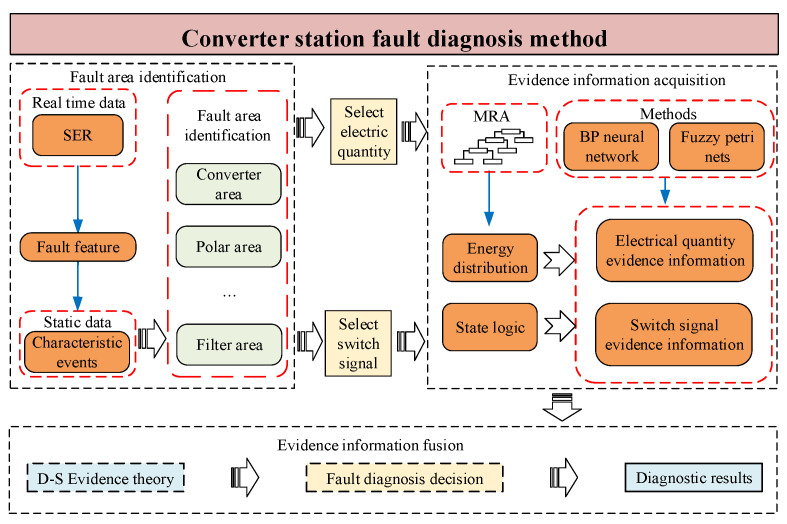
Converter station fault diagnosis method.

In this paper, an effective fault diagnosis method for converter stations is proposed, which possesses a step-by-step hierarchical structure and is divided into three steps: initial identification of the fault range, the acquisition of evidence information, and the fusion of evidence information. Figure 2 shows the overall flow of the method.

For fault range identification, preprocessing of the SER data is completed to obtain the characteristic events. Then, the correspondence between the characteristic events and the fault types in the historical SER data is determined. Finally, the fault area is determined according to the mapping relationship between each event and the fault type in the on-site SER event list. For evidence information acquisition, first, the state logic values of the switch signals and the energy distributions of the electrical waveforms after wavelet decomposition of the historical data are used as fault features to construct fuzzy Petri net and BP neural network models. The required electrical waveforms and switch signals in the on-site data are subsequently determined on the basis of the results of fault area identification. Finally, the electrical waveforms and switch signals are converted into fault features, and the evidence information is obtained via fuzzy inference and deep learning methods [19,20,21]. For evidence information fusion, first, the evidence inputs of electric and switch information are fused via D-S evidence theory. The fusion results are then analyzed to determine the final output and output diagnostic conclusions.

The basic theories involved in the above converter station fault diagnosis process are explained and illustrated in the following subsections.

### 2.1. Multi-Source Information Collection and Uploading Methods

Sensors are important front-end devices in SCADA systems, responsible for collecting on-site data. The SCADA network is a channel for data transmission, used to transfer the collected data from sensors and field devices to the central control system. Sensors play a crucial role in multi-source information transmission between the current main station system and the converter station. At present, active distribution network data collection and transmission are mostly achieved through SCADA networks. Due to the time interval of data collection in SCADA systems, transient data cannot be effectively collected in DC transmission systems.

Therefore, adopting a distributed data uploading scheme, different types of sensors are used to monitor and collect key information, and then, the data are transmitted to the main station system through specific protocols and uploading methods, as shown in Table 1.

1. Based on the DL/T 476 protocol, the SER event information collected by the DC control and protection device sensors is uploaded through the converter station event direct transmission server;
2. Based on the IEC 104 protocol, through the event direct transmission server of the converter station, the relevant teleindication, telemetering, and remote pulse status information of the converter station is uploaded, including the operation mode of the DC system, switch position, lock status, pole power, voltage, current, etc;
3. Based on the DL/T 860 protocol, the recording data collected by the DC control and protection device sensors are uploaded through the converter station control and protection recording direct transmission server;
4. Based on DL/T 860 and other protocols, relay protection information such as DC protection host settings are accessed through relay protection information substations.

As described above, by combining the characteristics and complementary features of multiple communication technology protocols, and through organic fusion, the main station system can obtain multi-source information on the converter station from the event direct transmission server, control and protection recording direct transmission server, and relay protection information substation, respectively. Therefore, multiple systems can leverage their respective advantages for more efficient information transmission.

### 2.2. Sequential Event Recording (SER) and Fault Range Identification Methods

The sequential event recording (SER) system of the converter station can record the real-time operation status of each device in the converter station and obtain important information such as event occurrence time, event level, event content, etc. Therefore, SER events contain a large amount of fault diagnosis information. The original SER data of the converter station contain six characteristic dimensions: time, host, system alarm, event level, alarm group, and event list. These are shown in Table 2.

Each SER dataset has multiple dimensions, but not all of them are favorable for fault diagnosis, and the SER dataset has problems such as large data volume, discrete feature information, and high redundancy, so SER preprocessing is needed. When a fault or state change happens at the converter station, the redundant system will generate the corresponding event set at the same time. This means that only keeping the data with a system alarm of A can greatly reduce the amount of data. The event level determines how important the SER event is for fault diagnosis and is divided into normal, triviality, alarm, and emergency. Keeping the SER data at the levels of alarm and emergency can get rid of a lot of information that is not useful.

The sequential event recording system will create different sets of SER events when the converter station is broken. All the events that are generated during a certain fault and are kept after SER preprocessing are often related to protection actions. Therefore, they are called characteristic events. The feature event database is generated by correlating statistical feature events with protective actions. The support count for each protection action is calculated based on the feature event database. Then, the calculated support counts are sorted to obtain the action status of protection and further infer the area where the fault occurred.

**Figure 3:**
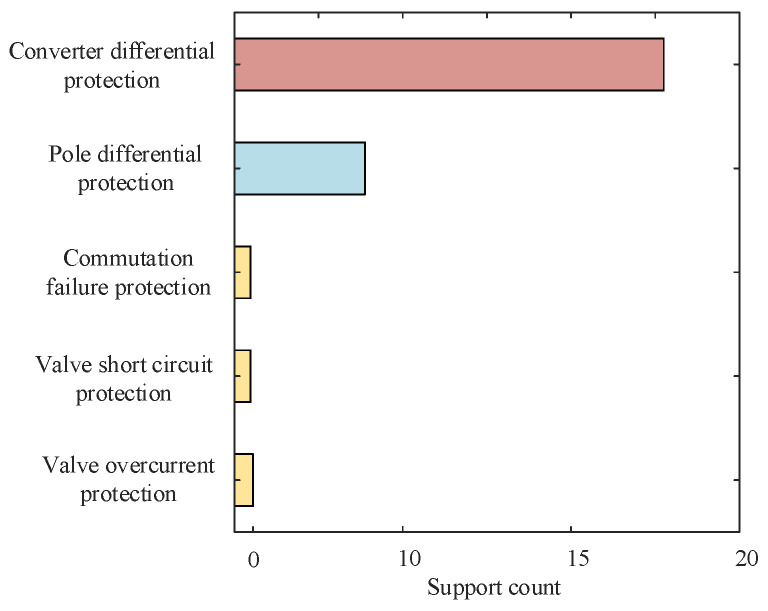
Analysis results of SER events.

Taking fault F3, which occurred at an actual project inverter station, as an example for analysis, the results are shown in Figure 3. Only the differential protection of the converter and the extreme differential protection have obvious counting results, indicating that the converter differential protection and the pole differential protection are in operation. The fault in the converter area can also cause the action of the pole differential protection, so the actions of the converter differential protection and the pole differential protection indicate that the fault occurred in the converter area. The preliminary determination of the fault area does not require accurate identification of the fault point, but rather, further selection of corresponding switch signals and electric quantities by obtaining the fault area. Therefore, it can be seen that in the first step of fault area identification, SER events can be used to narrow down the fault range from the entire system to a certain area.

### 2.3. The Method of Switch Signal Evidence Information Acquisition

A Petri net is a type of graphical modeling used to show how concurrent systems work. It has two main parts: places and transitions. Places represent the system’s states, and transitions show how it moves from one state to another. Places and transitions are connected by directed arcs [22]. Traditional Petri nets are based on Boolean logic, i.e., the value of each state can only be true or false, whereas fuzzy Petri nets allow states to take values between 0 and 1, indicating uncertainty or ambiguity. Compared with traditional Petri nets, fuzzy Petri nets can describe the behavior of a system in a more flexible way and yield better results in the fields of diagnosis and decision making [23,24].

**Figure 4:**
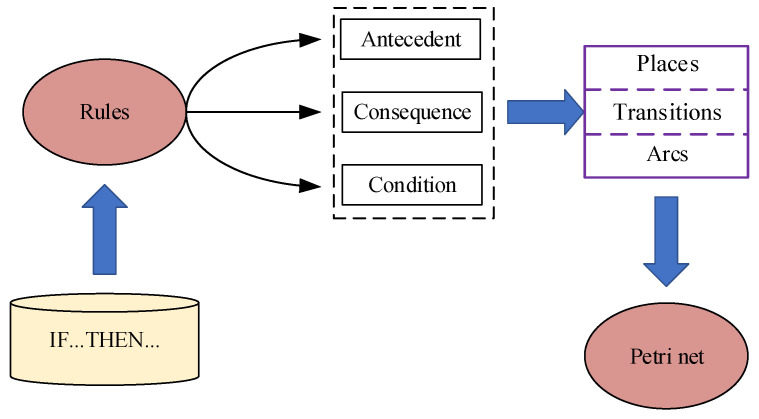
Petri net construction process.

The occurrence of a converter station fault is a typical discrete event dynamic system, that is, the system changes or generates events at a determined point in time. Therefore, Petri nets are a good way to describe the characteristics of converter station faults. By summarizing the main protection actions or waveform changes under different faults, rules can be formed, and a fault diagnosis Petri net for the converter station can be constructed. The network construction process is shown in Figure 4.

After obtaining the fuzzy Petri nets for the fault diagnosis of converter stations, the initial omen information of the faults should be substituted into the network for inference iteration to obtain the credibility of the occurrence of the faults and then obtain the Petri net evidence information. For example, the MYCIN confidence method is an excellent expert system inference method that can deal with uncertain information. This means that it can be used to improve the accuracy and credibility of fuzzy Petri nets’ inference results. The reasoning process of this method is as follows. First, three operators in the max-plus algebra are introduced in Equation (1): ⊕, ⊗, and a neg operator.

(1) $$\begin{array}{c} \left\{\begin{array}{c} a⊕b=c,c_{i}=\max\left(a_{i},b_{i}\right),\\ A⊗b=d,d_{i}=\max\left(a_{i1}\times b_{1},a_{i2}\times b_{2},\cdots ,a_{in}\times b_{n}\right),\\ \mathrm{neg}\left(b\right)=\left(1-b_{1},1-b_{2},\cdots 1-b_{n}\right), \end{array}\right. \end{array}$$

where *a*, *b*, *c* are m-dimensional vectors in the ⊕ operator, *A* is an *m* × *n* order matrix, *b* and *d* are *n*-dimensional and *m*-dimensional vectors in the ⊗ operator, respectively, and b is an m-dimensional vector in the neg operator.

Equation (2) is defined on the basis of the above operator:

(2) $$\begin{array}{c} \left\{\begin{array}{c} \;\mathrm{neg}\;\theta^{k}=1_{n}-\theta^{k}={\bar{\theta }}^{k},\\ \mu^{k}=I^{T}⊗\left(\;\mathrm{neg}\;\theta^{k}\right),\\ \sigma^{k}=\;\mathrm{neg}\;\left(\mu^{k}\right),\\ \theta^{k+1}=\theta^{k}⊕\left[\left(O\times U\right)⊗\sigma^{k}\right]. \end{array}\right. \end{array}$$

where *k* is the number of iterations; $1_{n}$ is an n-dimensional vector composed entirely of 1; $I^{T}$ is the transpose of the input matrix of the Petri net model; *O* is the output matrix of the Petri net model; *U* is a diagonal matrix consisting of the credibility of the fuzzy knowledge rules; $neg\theta^{k}$ is the credibility in the case of the places being negated, representing the degree of rejection of the rule by the fault omen information; $\mu^{k}$ and $\sigma^{k}$ represent the confidence when the premise of transition is true and false, respectively; $\theta^{k+1}$ is the degree of support for the rule by the fault omen information when the iteration is carried out up to k + 1 times; and the iteration is ended when $\theta^{k+1}$ = $\theta^{k}$.

**Figure 5:**
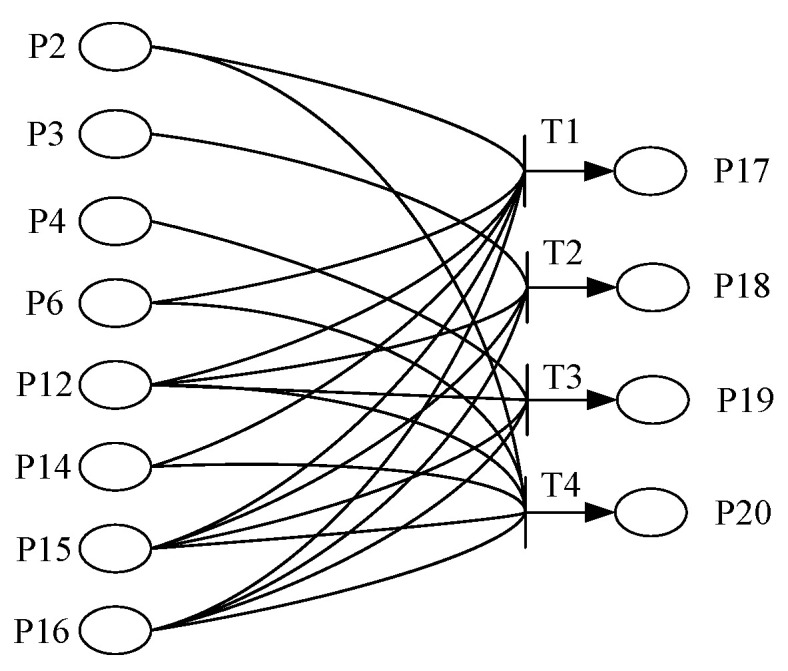
Fuzzy Petri net model for switch signal diagnosis of converter station.

The method of obtaining switch evidence information is still illustrated with a fault example. Firstly, the triggering situations of protection action signals and sequential control signals under various typical faults are summarized. Then, we obtain the corresponding Petri net model from the summarized switch signal rules. Finally, we use the switch signal-triggering situation of on-site faults as the input for iterative reasoning of the probability of each typical fault’s occurrence, that is, the switch quantity evidence information. Due to the large number of places and transitions in the overall Petri net, the network model is not easy to display in the main text. Therefore, only a portion of the switch signal Petri model will be presented, as shown in Figure 5. The Petri net places are defined as follows: P2: converter differential protection action; P3: Y-bridge valve short-circuit protection action; P4: D-bridge valve short-circuit protection action; P6: pole differential protection action; P12: X-locking; P14: Z-locking; P15: disconnect the AC circuit breaker; P16: pole isolation; P17: F3; P18: F4; P19: F12; P20: F11. This model is obtained via graphical modeling on the basis of the following four rules:

T1: IF P2 and P6 and P12 and P13 and P15 and P16, THEN P17

T2: IF P2 and P12 and P15 and P16, THEN P18

T3: IF P4 and P12 and P15 and P16, THEN P19

T4: IF P2 and P6 and P12 and P13 and P15 and P16, THEN P20

Among them, the commutation failure, due to its specificity, takes a confidence CF of rule S4 of 0.8, and the confidence of the rest of the rules is 0.9. The specificity of commutation failure refers to the fact that commutation failure is not a fault, but rather, a reflection of a control pulse transmission fault or other faults.

According to the above fuzzy Petri net model for the fault diagnosis of converter stations, the input matrix I and output matrix O of the network model can be obtained:

(3) $$I=\left\{\begin{array}{cccc} 1 & 0 & 0 & 1\\ 0 & 1 & 0 & 0\\ 0 & 0 & 1 & 0\\ 1 & 0 & 0 & 1\\ 1 & 1 & 1 & 1\\ 1 & 0 & 0 & 1\\ 1 & 1 & 1 & 1\\ 1 & 1 & 1 & 1\\ 0 & 0 & 0 & 0\\ 0 & 0 & 0 & 0\\ 0 & 0 & 0 & 0\\ 0 & 0 & 0 & 0 \end{array}\right\}\;O=\left[\begin{array}{cccc} 0 & 0 & 0 & 0\\ 0 & 0 & 0 & 0\\ 0 & 0 & 0 & 0\\ 0 & 0 & 0 & 0\\ 0 & 0 & 0 & 0\\ 0 & 0 & 0 & 0\\ 0 & 0 & 0 & 0\\ 0 & 0 & 0 & 0\\ 1 & 0 & 0 & 0\\ 0 & 1 & 0 & 0\\ 0 & 0 & 1 & 0\\ 0 & 0 & 0 & 1 \end{array}\right],$$

After it is clear that the fault occurred in the converter area, the corresponding switch signal of the converter area is called, and the protection action is as follows: commutation failure protection does not act, converter differential protection acts, Y-bridge valve short-circuit protection and D-bridge valve short-circuit protection do not act, valve over-current protection does not act, and protection in other areas does not act. The sequence control operations are as follows: X-locking, Z-locking, AC circuit breaker disconnection, and pole isolation. The initial value of the confidence level of the protection action is 0.9; otherwise, it is 0.1.

According to the above results, the evidence information for switch signal fault diagnosis can be obtained as follows: the confidence of the occurrence of faults F3 and F11 are 0.81, the confidence of fault F5 is 0.153, the confidence of fault F7 is 0.144, and the confidence of the other faults is very small. These results indicate that the fault has a higher probability of occurring at positions F3 and F11, and a lower probability of occurring at positions F5 and F7. Due to the similar fuzzy knowledge rules of F3 and F11 in Petri nets, the iterative reasoning results also have similar confidence results. This indicates that fault diagnosis based on switch signals can further narrow down the fault range from the entire region to several undetermined faults. However, the problem of an inability to accurately locate the fault point still exists, and it is necessary to combine other sources of information for a comprehensive diagnosis.

### 2.4. The Method of Electrical Quantity Evidence Information Acquisition

In fact, similar to the acquisition of switch signal evidence information, electrical quantity evidence information can also be obtained through expert inference systems. However, the changes in electrical quantities are often complex and rapid and may even exhibit periodic variations. When there is a large amount of historical data, problems such as Petri nets being too redundant and difficulty in describing waveform variations can arise. Therefore, this method is not suitable for large-scale fault diagnosis. This article adopts a BP neural network construction method based on wavelet energy distribution, which can effectively obtain electric evidence information even when facing complex transient waveforms.

The purpose of multiscale decomposition is to construct a set of standard orthogonal bases in the square producible space that can infinitely approximate any function in the space, and the details of the original signal can be obtained [25,26]. The essence of wavelet decomposition is the multiscale decomposition of the detected signals, and the normal operating state and fault state signals of the frequency band components are different. Wavelet transform through multiresolution analysis can be achieved for the purpose of time–frequency analysis through the capture of fault information to achieve fault feature extraction [27,28]. Faults at converter stations are accompanied by changes in direct current, and direct current transient signals can generally reflect changes in key electrical information such as the trigger angle and direct voltage. Therefore, performing a wavelet transform on the DC current can reflect the differences in different faults. However, wavelet analysis cannot quantify fault characteristics and is difficult to use directly for fault diagnosis. In this work, we consider quantifying fault characteristics via wavelet coefficients. The difference in wavelet energy in each layer of the DC current accurately reveals the fault characteristics.

The wavelet coefficients at different scales can be obtained via wavelet transform. According to Parseval’s principle, the wavelet energy $E_{f}$ of the signal is related to the wavelet coefficients at each scale as follows:

(4) $$\begin{array}{cccc} & & E_{f}=\int {\left|f\left(t\right)\right|}^{2}dt=\sum_{k}{\left|a_{J}\left(k\right)\right|}^{2}+\sum_{j=1}^{J}\sum_{k}{\left|d_{j}\left(k\right)\right|}^{2}, & \end{array}$$

(5) $$\begin{array}{cccc} & & E_{f}=E_{a_{J}}+\sum_{j=1}^{J}E_{d_{j}}, & \end{array}$$

The low-frequency energy EaJ at the *J*-th scale and the high-frequency energy EdJ at the *j*-th scale can be further obtained as follows:

(6) $$\begin{array}{cccc} & & E_{a_{J}}=\langle a_{J}\left(k\right),a_{J}\left(k\right)\rangle ={∥a_{J}∥}_{2}^{2}, & \end{array}$$

(7) $$\begin{array}{cccc} & & E_{d_{j}}=\langle d_{j}\left(k\right),d_{j}\left(k\right)\rangle ={∥d_{j}∥}_{2}^{2}. & \end{array}$$

**Figure 6:**
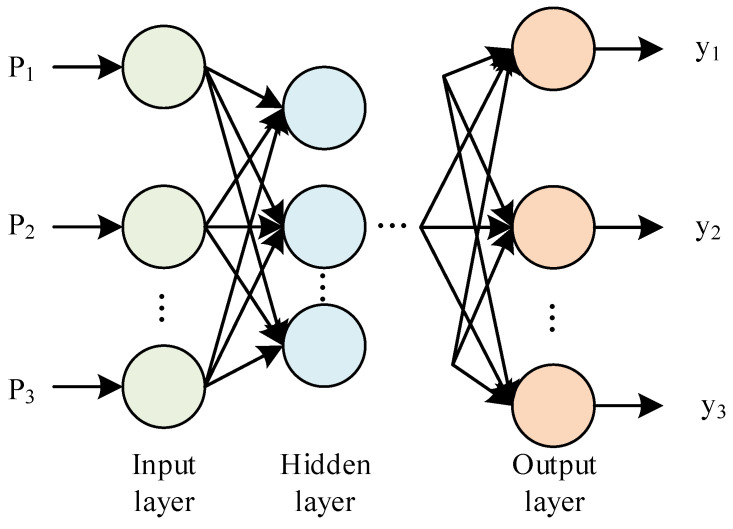
BP neural network structure diagram.

Normalizing and expressing the faulty recorded signals in terms of energy at different scales is carried out. Then, by calculating the energy levels at each scale, it is possible to determine the characteristics of the signals in various frequency bands. Deep learning is then performed on the energy features to correctly identify different fault signals. BP neural networks have more desirable results than other deep learning methods when dealing with the mapping of low-dimensional feature information spaces to fault space situations. Therefore, in this paper, BP neural networks are used to obtain information about electrical quantity evidence. A BP neural network is usually composed of three layers, i.e., an input layer, a hidden layer, and an output layer. A typical three-layer BP network structure is shown in Figure 6; the network is arranged in layers, and only adjacent layers are linked with network weights. The hidden layer of the network mostly adopts a sigmoid function as the excitation function, and the output layer mostly adopts a linear function.

Determining a BP network generally requires several considerations, such as the number of layers in the network, the number of neurons in each layer, and the activation function [29]. For a specific problem, if the input and output variables have been determined, the number of nodes in the input and output layers of the network is also determined. Taking the fault range [F3, F5, F7, F11] as an example, the number of input layer nodes in the neural network is equal to the dimension of the input vector, and the number of output layer nodes is equal to the number of categories to be predicted. Therefore, the number of input layer nodes in the network is 5, and the number of output layer nodes is 4. The key to determining the BP neural network is to determine the number of layers in the hidden layer and the number of nodes in each layer. Increasing the number of layers can further reduce the error and improve the accuracy while simultaneously increasing the training time of the network weights. The preliminary selection of one hidden layer is sufficient to meet the requirements while avoiding the complexity of the network.

**Figure 7:**
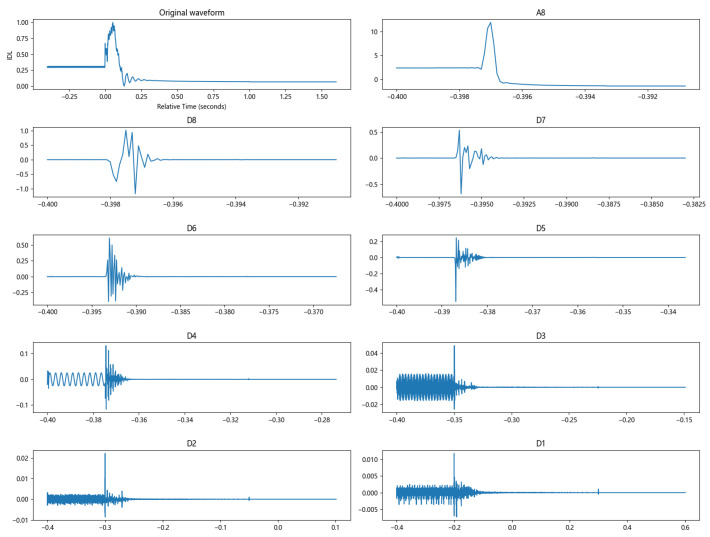
Results of 8-layer wavelet decomposition of direct current.

**Figure 8:**
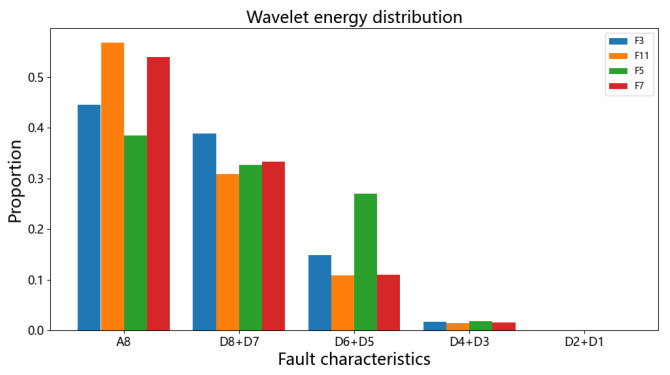
Wavelet energy distribution condition.

The Daubechies wavelet has tight support; among them, db8 is more suitable for analyzing such short and fast transient signals and can better reflect different fault characteristics, so the db8 wavelet is chosen for multiscale decomposition. Figure 7 shows the results of the eight-layer wavelet decomposition of the direct current at the time of the high-end grounding of the inverter station. Through wavelet decomposition, which clearly reveals the occurrence of the mutation signal, at the moment of signal mutation, the layers and high-frequency and low-frequency components are relatively rich. The wavelet decomposition is carried out by taking the DC current of the converter station under three different fault conditions as an example. In the actual calculation, the low-frequency and medium-frequency energy are taken as A8. Considering that the high-frequency energy of adjacent layers is relatively similar, (D8 + D7), (D6 + D5), (D4 + D3), and (D2 + D1) are combined to obtain the high-frequency wavelet energy at each scale. The energy ratios of different frequency bands are used as indicators to reflect the difference in direct current in different transient processes, and the distribution of wavelet energies is obtained after normalization, as shown in Figure 8. The figure and table show that different fault types have significantly different energy proportions, so the wavelet energy distribution of the direct current can be used as a feature input to the BP neural network for training.

### 2.5. D-S Evidence Theory

Dempster–Shafer evidence theory (D-S evidence theory) is a theoretical model for reasoning and decision making that aims to explain how evidence is handled, and is utilized in the reasoning decision-making process. In classical D-S evidence theory, many mutually exclusive propositions form an identification framework ⊖, and the propositions within the framework are assigned a basic probability assignment (BPA), which is also known as the mass function, to obtain the importance of each proposition within the framework [30,31,32]. Assuming proposition A, the mass function satisfies the following conditions:

(8) $$\begin{array}{cccc} & & \left\{\begin{array}{c} m:2^{⊖}\to \left[0,1\right],\\ m(⌀)=0,\\ \sum_{A\subseteq \Theta }m\left(A\right)=1, \end{array}\right. & \end{array}$$

where $A\subseteq 2^{⊖}$ is a proposition, *m* is the mass function of the proposition, and m(A) denotes the magnitude, i.e., the importance, of the mass of proposition *A*.

Assuming that the basic probabilities of two mutually independent pieces of evidence information are denoted as $m_{1}$ and $m_{2}$, new probability assignments can be obtained via the D-S evidence theory fusion rule:

(9) $$\begin{array}{cccc} & & m\left(A\right)=\left[m_{1}+m_{2}\right]\left(A\right)=\left\{\begin{array}{cc} 0, & \;A=\Theta ,\\ \frac{\sum_{A_{i}\cap B_{j}=A}m_{1}\left(A_{i}\right)m_{2}\left(B_{j}\right)}{1-\sum_{A_{i}\cap B_{j}=⌀}m_{1}\left(A_{i}\right)m_{2}\left(B_{j}\right)}, & A\neq \Theta . \end{array}\right. & \end{array}$$

where m(A) is defined as the orthogonal sum of $m_{1}$ and $m_{2}$, representing the probability that proposition *A* occurs after the fusion of evidential information.

Since the obtained converter station’s electrical and switching evidence information are probability values located in the interval [0, 1], after fusion, only normalization is needed to obtain the basic probability distribution of the diagnostic output results in a certain interval on the target, and the two are completely independent of each other as evidence information. There is no conflict of evidence information in the fusion of information, which can lead to more accurate and credible conclusions. Therefore, the use of the D-S method has a better effect when applied to the fusion of fault diagnosis evidence information of the converter station [32,33,34].

## 3. Experiments and Results

### 3.1. Relevant Experimental Background

The data used in this article come from the real-time digital simulation (RTDS) system of a certain inverter station. This article ensures the diversity and comprehensiveness of data by setting different operating modes and transition resistors, and constructs Petri net models and trains BP neural networks using simulated data.

**Figure 9:**
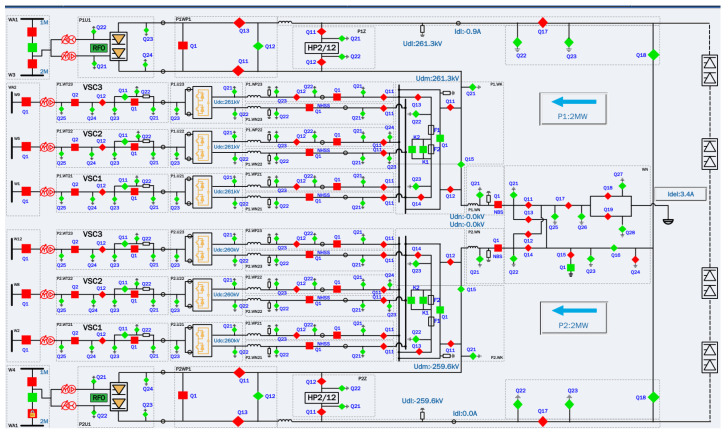
Diagram of RTDS simulation model of DC field structure.

In essence, RTDS is a parallel computer system specially designed for the real-time simulation of the transient process of power systems. Most of the commonly used simulation tools are non-real-time simulation programs. For these simulators running on digital computers, the limitation is that it takes many minutes or even hours to calculate the response of the HVDC simulation system within a few seconds of failure. This non-real-time simulation speed cannot meet the needs of real-time interactive testing with external physical control devices and protection devices. The RTDS simulation system can solve the above problems well and simulate the protection action of and waveform change in the HVDC system after a fault. Figure 9 shows a DC field structure diagram of the RTDS simulation model of the HVDC transmission system.

### 3.2. Evidence Information Acquisition Results and Analysis

The on-site data of different faults are used as samples to verify the feasibility of the evidence information acquisition method proposed in this paper, and the results are shown in Table 3 and Table 4. To reliably and accurately identify faults, the confidence is set to greater than 0.8 to confirm the occurrence of faults. For the switch evidence information, the expert inference system can effectively obtain the switch evidence information, but the confidence requirement of fault diagnosis cannot be met with only this evidence information. Due to the similar or even identical protection and sequential control operations of these faults, there are issues such as misjudgments and insufficient confidence in the diagnostic results based on switch evidence information. For the electric evidence information, the evidence information obtained by the BP neural network has a higher confidence level than that obtained by the inference system, but it still cannot achieve the diagnosis of all samples. This finding indicates that only a single piece of evidence cannot be used to reliably identify faults.

### 3.3. Evidence Information Fusion Results and Analysis

The fusion results of the evidence information and their diagnostic conclusions are shown in Table 5, where A and B represent the switch signal Petri evidence information and the electrical quantity BP neural network evidence information, respectively. The confidence values of the four kinds of engineering practical fault diagnosis results after evidence information is obtained are 0.9296, 0.9542, 0.9786, and 0.9008. The confidence of the diagnosis results is closer to 1 than before fusion. This indicates that evidence information fusion reduces the degree of influence of misinformation on the results and improves the completeness of the information and the reliability of the diagnostic results. The diagnostic results of the samples are all consistent with the actual occurrence of the faults, which verifies the feasibility and correctness of the diagnostic method of using evidence information fusion in DC converter stations.

### 3.4. Comparison with Other Methods

To verify the superiority of the method proposed in this paper, several classical fault diagnosis methods from other papers are selected, such as the expert system [10] and BPN [15]. The results are then compared with those of the method proposed in this paper, and the results are shown in Table 6 and Table 7. Table 6 shows that, compared with the other methods, the method proposed in this paper can obtain fault diagnosis results with higher confidence. Therefore, its diagnostic reliability is significantly better than that of the other methods. Table 7 shows that compared with the other methods, the method proposed in this paper has higher diagnostic accuracy when facing the same type and quantity of sample sets. In summary, the method proposed in this paper has significant advantages compared to traditional diagnostic methods.

## 4. Discussion

At present, there are few related studies on converter station information fusion and diagnosis, and the method proposed in this paper can effectively assist operation and maintenance personnel in diagnosing typical converter station faults. Theoretically, if there are enough historical data of typical faults in the DC transmission system, a comprehensive evidence information acquisition model can be constructed to achieve fault diagnosis of the whole DC system. However, in reality, the control and protection devices of the HVDC transmission system are multilayered and complex. Therefore, it is still a challenge to carry out in-depth research on evidence information acquisition and fusion from the perspective of the whole DC system.

## 5. Conclusions

This paper proposes a new fault diagnosis method for converter stations. First, on the basis of the preprocessing of converter station SER data and the statistics of SER characteristic events, preliminary identification of the converter station fault area is achieved, and the fault point range is obtained, which lays the foundation for the subsequent fault diagnosis and analysis. Second, the fuzzy Petri net and BP neural network models for fault diagnosis of the converter station are constructed, and the corresponding evidence information is obtained. Finally, fusion of the switch signal evidence information and the electrical quantity evidence information of the converter station is completed, and accurate identification of the fault point of the converter station is achieved according to the fusion results.

These results show that single-evidence information leads to problems such as low reliability of the diagnostic results and incomplete diagnostic scope, whereas all the diagnostic results based on the fusion of evidence information are able to reliably and accurately determine the point of fault.

## Figures and Tables

**Table 1:** Multi-source information upload scheme.

Information Type	Information Collection and Uploading
Event information	Protection device sensors are transmitted through SCADA
Protection action	Protection device sensors are transmitted through SCADA
Telemetry	Protection device sensors are transmitted through SCADA
Protection setting	Transmitted through protection information substation
Wave recording	Send the protection device to the file server
Other information	Transmitting information through wireless networks

**Table 2:** SER data feature dimension.

ID	SER Feature Type	Feature Type Content
1	Time	The Time of the occurrence of the event (accurate to milliseconds)
2	Host	The control and protection device server that generates the event
3	System alarm	Redundant systems for SER (A/B)
4	Event level	The operational status level of the event
5	Alarm group	The control device or component that generates the event
6	Event list	A detailed description of the event content

**Table 3:** Diagnostic results of switch signal Petri evidence information of converter station.

Sample	Network Output				Actual Fault	Diagnosis
	F3	F5	F7	F11		
1	0.81	0.153	0.144	0.81	F3	indefinite
2	0.252	0.855	0.189	0.171	F5	F5
3	0.369	0.126	0.648	0.153	F7	indefinite
4	0.81	0.153	0.144	0.81	F11	indefinite

**Table 4:** Diagnostic results of elctric quantity BP neural network evidence information of converter station.

Sample	Network Output				Actual Fault	Diagnosis
	F3	F5	F7	F11		
1	0.8656	0.0695	0.0153	0.0497	F3	F3
2	0.0216	0.8147	0.0001	0.1637	F5	F5
3	0.0039	0.0013	0.9201	0.0747	F7	F7
4	0.0347	0.235	0.0047	0.7256	F11	indefinite

**Table 5:** Fusion of switch signal Petri and electrical quantity BP neural network evidence information and its diagnostic results in converter stations.

Sample		Network Output Normalization				Fusion Results				Diagnosis
		F3	F5	F7	F11	F3	F5	F7	F11	
1	A	0.4225	0.0798	0.0751	0.4225	0.9296	0.0141	0.0029	0.0534	F3
	B	0.8656	0.0695	0.0153	0.0497					
2	A	0.1718	0.5828	0.1288	0.1166	0.0075	0.9542	0 .0000	0.0384	F5
	B	0.0216	0.8147	0.0001	0.1637					
3	A	0.2847	0.0972	0.5000	0.1181	0.0024	0.0003	0.9786	0.0188	F7
	B	0.0039	0.0013	0.9201	0.0747					
4	A	0.4225	0.0798	0.0751	0.4225	0.0431	0.0551	0.0010	0.9008	F11
	B	0.0347	0.2350	0.0047	0.7256					

**Table 6:** Comparison of confidence between proposed method and traditional methods.

Sample	Confidence of Diagnostic Results (%)		
	Expert System	BPN	Proposed Method
1	81	86.56	92.96
2	85.5	81.47	95.26
3	64.8	92.01	97.86

**Table 7:** Comparison of accuracy between proposed method and traditional method.

Sample	Accuracy of Diagnostic Results (%)	
	BPN	Proposed Method
1	63.64	98.70
2	89.61	97.40
3	53.25	92.21

## Data Availability

The data used to support the findings of this study are available from the corresponding author upon request.

## Abbreviations

The following abbreviations are used in this manuscript:

SER	Sequential event recording
RTDS	Real-time digital simulation
HVDC	High-voltage direct current
BP	Back-propagation
